# A pilot randomized controlled trial evaluating outdoor community walking for knee osteoarthritis: walk

**DOI:** 10.1007/s10067-022-06477-5

**Published:** 2023-01-24

**Authors:** S. J. J. Drummen, S. Balogun, A. Lahham, K. Bennell, R. S. Hinman, M. Callisaya, G. Cai, P. Otahal, T. Winzenberg, Z. Wang, B. Antony, I. P. Munugoda, J. Martel-Pelletier, J. P. Pelletier, F. Abram, G. Jones, D. Aitken

**Affiliations:** 1 Menzies Institute for Medical Research, University of Tasmania, Hobart, TAS 7000 Australia; 2 Australian National University, Canberra, Australia; 3 Monash University, Melbourne, Australia; 4 The University of Melbourne, Melbourne, Australia; 5 Department of Epidemiology and Biostatistics, School of Public Health, Anhui Medical University, Hefei, Anhui China; 6 Osteoarthritis Research Unit, University of Montreal Hospital Research Centre (CRCHUM), Montreal, QC Canada; 7 Medical Imaging Research & Development, ArthroLab Inc, Montreal, QC Canada

**Keywords:** Aerobic, Exercise, Joint, MRI, Structure

## Abstract

**Objectives:**

To determine the feasibility of a randomized controlled trial (RCT) examining outdoor walking on knee osteoarthritis (KOA) clinical outcomes and magnetic resonance imaging (MRI) structural changes.

**Method:**

This was a 24-week parallel two-arm pilot RCT in Tasmania, Australia. KOA participants were randomized to either a walking plus usual care group or a usual care control group. The walking group trained 3 days/week. The primary outcome was feasibility assessed by changes being required to the study design, recruitment, randomization, program adherence, safety, and retention. Exploratory outcomes were changes in symptoms, physical performance/activity, and MRI measures.

**Results:**

Forty participants (mean age 66 years (SD 1.4) and 60% female) were randomized to walking (*n* = 24) or usual care (*n* = 16). Simple randomization resulted in a difference in numbers randomized to the two groups. During the study, class sizes were reduced from 10 to 8 participants to improve supervision, and exclusion criteria were added to facilitate program adherence. In the walking group, total program adherence was 70.0% and retention 70.8% at 24 weeks. The walking group had a higher number of mild adverse events and experienced clinically important improvements in symptoms (e.g., visual analogue scale (VAS) knee pain change in the walking group: − 38.7 mm [95% CI − 47.1 to − 30.3] versus usual care group: 4.3 mm [− 4.9 to 13.4]).

**Conclusions:**

This study supports the feasibility of a full-scale RCT given acceptable adherence, retention, randomization, and safety, and recruitment challenges have been identified. Large symptomatic benefits support the clinical usefulness of a subsequent trial.

**Trial registration number:**

12618001097235.

**Table:** 

**Key Points** *• This pilot study is the first to investigate the effects of an outdoor walking program on knee osteoarthritis clinical outcomes and MRI joint structure, and it indicates that a full-scale RCT is feasible.* *• The outdoor walking program (plus usual care) resulted in large improvements in self-reported knee osteoarthritis symptoms compared to usual care alone.* *• The study identified recruitment challenges, and the manuscript explores these in more details and provides recommendations for future studies.*

**Supplementary Information:**

The online version contains supplementary material available at 10.1007/s10067-022-06477-5.

## Introduction

Knee osteoarthritis (KOA) is a leading and increasingly prevalent cause of disability [1]. The development of this heterogeneous disorder involves inflammatory, mechanical, and metabolic factors, and altered pain processing at the joint and brain levels [2]. Physical treatments (including therapeutic exercise, education, and hyaluronic acid or corticosteroid joint injections) have a substantial effect on improving pain, functional status, and inflammatory markers [1, 3–5]. However, general practitioners commonly prescribe medications and refer patients to orthopedic surgeons, even for new osteoarthritis-related problems [3]. Exercise is recommended as a cornerstone treatment of KOA [1], yet there is a gap in the implementation of this recommendation [3, 6].

The reasons for the lack of uptake are complex but include misconceptions that exercise causes joint harm [7]. However, the available evidence to refute this belief has important limitations. Magnetic resonance imaging (MRI) studies allow greater insight into structural adaptations that may occur [2]. A recent systematic review of MRI randomized controlled trials (RCTs) investigating the impact of knee joint loading exercise on articular cartilage concluded that exercise seems to not be harmful for articular cartilage [8]. A separate systematic review concluded that exercise therapy did not change cartilage morphology or synovitis/effusion but may slightly increase the likelihood for bone marrow lesion (BML) worsening [9]. The limitations of studies to date include small sample sizes, variable adherence to exercise (4–70%), inadequate reporting of participant withdrawal, and the use of exercises that may place too little (e.g., aquatic) or excessive (e.g., high-impact) load on a knee to induce beneficial joint structure changes [8, 9].

Outdoor walking is cost efficient, practical, and easily reproduced. Previous research has shown that walking can be used to reduce KOA-related pain and functional restrictions, but evidence for its potential effects on joint structure is contradictory [8–12]. Some studies show a detrimental effect [10–12], while others show either no effect or a beneficial effect [8, 9]. The structural effects of walking have not yet been investigated by an adequately powered MRI RCT. This pilot RCT aimed to determine the feasibility of an RCT examining outdoor community walking on KOA symptoms and MRI knee structural change to inform the design of future large-scale studies.

## Materials and methods

WALK was a pilot single-center two-arm RCT conducted in Tasmania, Australia. The trial was registered on the Australian New Zealand Clinical Trials Registry prior to recruitment (12,618,001,097,235) and is reported according to the CONSORT 2010 extension to randomized pilot and feasibility trials [13]. Ethics approval was from the Tasmanian Health and Medical Human Research Ethics Committee (H0017108), and all participants provided written informed consent.

### Recruitment and screening

Recruitment and screening took place from October 2018 to June 2019. Participants were recruited from the community local and social media advertisements. All potential participants were pre-screened via telephone followed by face-to-face screening, during which a detailed explanation of the project was given and written informed consent was obtained. Participants were eligible if they were aged 45 years or over, met the American College of Rheumatology (ACR) criteria for KOA [14] by clinical diagnosis, had symptomatic KOA for at least 24 weeks, had a pain visual analogue scale (VAS) score of at least 40 mm/100 mm over the last 7 days, and a BML present on MRI. Additional eligibility criteria include no difficulty in walking a city block (75–100 m) and willingness to participate in a walking program for 24 weeks with the ability to attend the scheduled walking classes. Excluded were individuals with any of the following: another form of arthritis, significant trauma to the study knee in the previous 12 months, receiving intra-articular therapy during the previous 24 weeks, were experiencing severe knee pain (> 80 mm VAS) while standing, were using a gait aid, planned to commence new forms of exercise, or undergo knee or hip surgery in the next 24 weeks. Analgesic medications were recorded, although no restrictions were made. Individuals with any conditions that precluded safe participation in exercise (e.g., a heart condition), as assessed by the adult pre-exercise screening tool (stage 1) [15], were required to receive medical clearance from their general practitioner before enrolling. Participants had to have at least one eligible knee, determined by verbal screening, clinical examination, and an MRI scan. When a participant had two eligible knees, the knee with the worst pain was selected as the study knee.

### Randomization

Eligible participants were randomized with a 1:1 ratio to either *outdoor community walking plus usual care* (walking group) or *a usual care control group* for 24 weeks. Allocation of participants to either the walking group or usual care was based on simple randomization using computer-generated random numbers via a central randomization website hosted by the University of Tasmania. This was conducted by a blinded staff member with no direct involvement in the study. In this study the assessors, MRI readers were blinded to treatment allocation, but participants were not.

### Outdoor community walking plus usual care group (walking group)

The participants who were randomized to the walking group were asked to train 3 days per week for 24 weeks. Each week consisted of two sessions in a supervised group and one unsupervised session at a location of their choice. The walking program was based on a protocol published by Ettinger et al. [16]. Each session (supervised or unsupervised) lasted 1 h and consisted of a 10 min warm-up (Appendix 1), a 40-min walk aimed at 50–70% of heart rate reserve (using the validated Borg rating of perceived exertion scale (RPE)) [17], and a 10-min cool-down consisting of slow walking and 3 flexibility exercises (Appendix 1). Each supervised class was led by one trainer who was either a physiotherapist or exercise physiologist. The participants reported adherence to their unsupervised sessions and provided feedback via an online questionnaire which was sent by email each week. To stimulate adherence, participants had flexibility to choose day/time/location/trainer, were allowed to bring family members on walks, and received a Fitbit on study completion. In addition, trainers would facilitate social support, positive reinforcement, goal setting, rewards for attendance, frequent contact, and recognition in study update newsletters.

All study staff (trainers and research assistants) participated in a half-day workshop for protocol training, which included instructions about administering the program, monitoring adherence, and education about behavioral change [18]. Additionally, the trainers attended an on-site introduction to the three outdoor group walk locations where optional walking back, early opt-out, and loops options were shown. By using these options, the intensity and/or distance of the walk could be tailored to each participant. Adequate exercise intensity was guided by trainers who used the RPE. The walking group also received the same care that was provided to the usual care group, as outlined below.

### Usual care group

Participants in the usual care group received generic information about KOA and community services and resources (Arthritis & Osteoporosis Tasmania resources, Arthritis Australia flyer, and information about the website “MyJointPain” and a local physiotherapy program for KOA patients). Participants in the usual care group were discouraged from initiating any new exercise program for the 24-week study duration. Uptake of new activity was assessed by a questionnaire and objectively with accelerometers at screening, 12 and 24 weeks. Participants in the usual care group were given a Fitbit upon study completion to encourage retention.

### Safety

All adverse events (AEs) were recorded. A research assistant determined in communication with the participant whether an adverse event was probably not, possibly related or probably related to the study. Furthermore, the NRS-11 [19] was used to measure pain before and after each group walking session, as a way to monitor acute pain exacerbations. Pain was accepted during walking but monitored. An increase in pain from pre-walk levels of 0 to 2 was considered safe, from 3 to 5 acceptable, and increases above 5 were flagged as high risk [20].

### Retention

If participants withdrew from the study before 24 weeks, the reason and date were recorded. Missed walks from these participants were considered in the adherence counts until the official withdrawal date.

### Primary outcome

The primary outcome will indicate whether the study protocol is feasible by assessment of *design* (any required changes to the protocol during the pilot), *recruitment and screening* (duration and number of people screened to enroll 48 participants; this target was chosen to enable the estimation of effect sizes that are small to large [21]), *randomization* (balance of characteristics in each group), *adherence* (number and percentage of supervised sessions, unsupervised sessions and total sessions that were completed), *safety* (number and description of AEs by group), and *retention* (number of participants that withdrew by group).

### Exploratory outcomes

Exploratory outcome measurements were included to ensure that feasibility was assessed with participants undertaking a study protocol as similar to a large-scale trial as possible. Symptoms were measured using visual analogue scale (VAS) knee pain (0–100 mm), Western Ontario and McMasters Universities Osteoarthritis Index (WOMAC) knee pain (0–500 mm), WOMAC knee function (0–1700 mm), WOMAC knee stiffness (0–200 mm) (lower is better), and Osteoarthritis Research Society International-Outcome Measures in Rheumatology Clinical Trials (OARSI-OMERACT) [22] response to treatment. Isometric leg strength (predominantly quadriceps and hip extensors) was assessed simultaneously for both legs in kilogram (kg) by dynamometry (TTM Muscular Meter Tokyo, Japan). Physical performance was measured as indicated by OARSI [23], including the 30-s chair stand test, 40-m fast-paced walk test (m/s) and 6-min walk test (higher scores are better), and the timed up and go test and stair climb test (lower scores are better). Physical activity was assessed by waist-worn ActiGraph® wGTX3-BT (Firmware 1.9.2) activity monitors (ActiGraph LLC, Fort Walton Beach, FL, USA) if participants had at least 4 days of 10 h wear time, using settings as recommended by Migueles et al. [24]. Health-related quality of life and utility was assessed using the assessment of quality of life (AQoL-8D) [25] (0–1) and the EuroQol 5-dimension 5-level (EQ-5D-5L) [26] (0–1) questionnaires (1 = full health). Depression was assessed using the patient health questionnaire (PHQ-9) [27] (0–27) (lower is better).

### MRI knee pathology

An MRI scan of the “study” knee was acquired with a 1.5 T whole-body magnetic resonance unit (GE Optima 450 W, Milwaukee, USA) using a dedicated 8-channel knee coil at weeks 0 and 24. Image sequences included (1) a T1-weighted fat-saturated 3D gradient-recalled acquisition and (2) proton density fat-saturated 2D fast spin echo sequence. The parameters are described in Appendix 2.

BMLs were assessed on the proton density-weighted sequences and defined as areas of increased signal adjacent to the subcortical bone at the medial tibial (anterior and posterior), medial femoral (anterior and posterior), lateral tibial (anterior and posterior), lateral femoral (anterior and posterior), superior patella, and inferior patella sites, by measuring the maximum area of the lesion (mm^2^) as previously described [28]. Intra-observer repeatability was assessed in 20 participants with at least 1-week interval between the two readings, with an intra-class correlation coefficient (ICC) ranging from 0.86 to 0.98.

Effusion-synovitis was defined as the presence of intra-articular fluid-equivalent signal on the proton density-weighted images. The volume of effusion-synovitis [29] was measured using semi-automated segmentation, and the final 3D volume rendering was generated using a free open-source imaging software (3D Slicer, version 4.10, National Alliance of Medical Image Computing, NA-MIC). The ICC for intra- and inter-observer repeatability for this method has been previously reported as 0.99 and 0.84, respectively [29].

Cartilage defects were assessed using a modified outer bridge system [28] at the medial tibial, medial femoral, lateral tibial, lateral femoral, and patella sites. Grading ranged from grades 0 (normal cartilage) to 4 (full-thickness chondral wear) as previously described [28]. ICCs for intra- and inter-observer repeatability ranged from 0.94 and 0.93 for the total score of cartilage defects, as previously described [28].

Meniscal extrusions were scored separately at the anterior, middle, and posterior horns (medially/laterally). The intra- and inter-reader ICCs ranged from 0.85 to 0.92 for meniscal extrusions, as previously described [30].

### Additional measures

The following were also assessed: (1) *demographics:* sex and date of birth; (2) *anthropometrics:* height and weight to calculate BMI and waist and hip circumference; (3) *standing anteroposterior semi-flexed X-ray of the study knee:* from this image, static knee alignment from the anatomic axis based upon the methods of Moreland et al. [31] and radiographic KOA severity was measured by consensus with two readers (GJ, SD) utilizing the OARSI atlas to grade osteophytes and joint space narrowing (JSN) [32]. Intra-observer reproducibility was assessed for 20 participants, the ICC (2-way mixed-effects model) of the measurements was 0.95 for knee alignment, 0.84 for osteophytes and 0.93 for JSN; (4) *participant satisfaction with the walking intervention:* this was self-reported by participants on a scale of 1 (extremely unsatisfied) to 7 (extremely satisfied) at week 24.

### Statistical analyses

Analyses were performed using Stata (version 16; StataCorp, College Station, TX, USA) and intention-to-treat with all available data from all randomized participants using their randomized group allocation. Descriptive statistics were used to summarize the program adherence, the adverse events, and the randomization of characteristics in groups at baseline. Changes in exploratory outcomes were analyzed to provide an indication of the direction and magnitude of changes in symptoms and physical performance/activity and whether there may be some indication of MRI structural change. Continuous outcomes at baseline and 24 weeks are shown as means and standard errors derived from unadjusted linear mixed-effects models. The change between these time points by group is presented as means and 95% CI. To investigate whether there was a difference in slope between both groups, the difference of differences was analyzed by linear mixed-effects models for treatment, time point, and a treatment/time point interaction, adjusted for sex and age as covariates. The models were adjusted for the baseline value of the corresponding outcome, which is considered best practice in RCT analysis [33]. For the analyses of categorical variables, log-binomial regression was used.

## Results

### Primary results

**Fig. 1:**
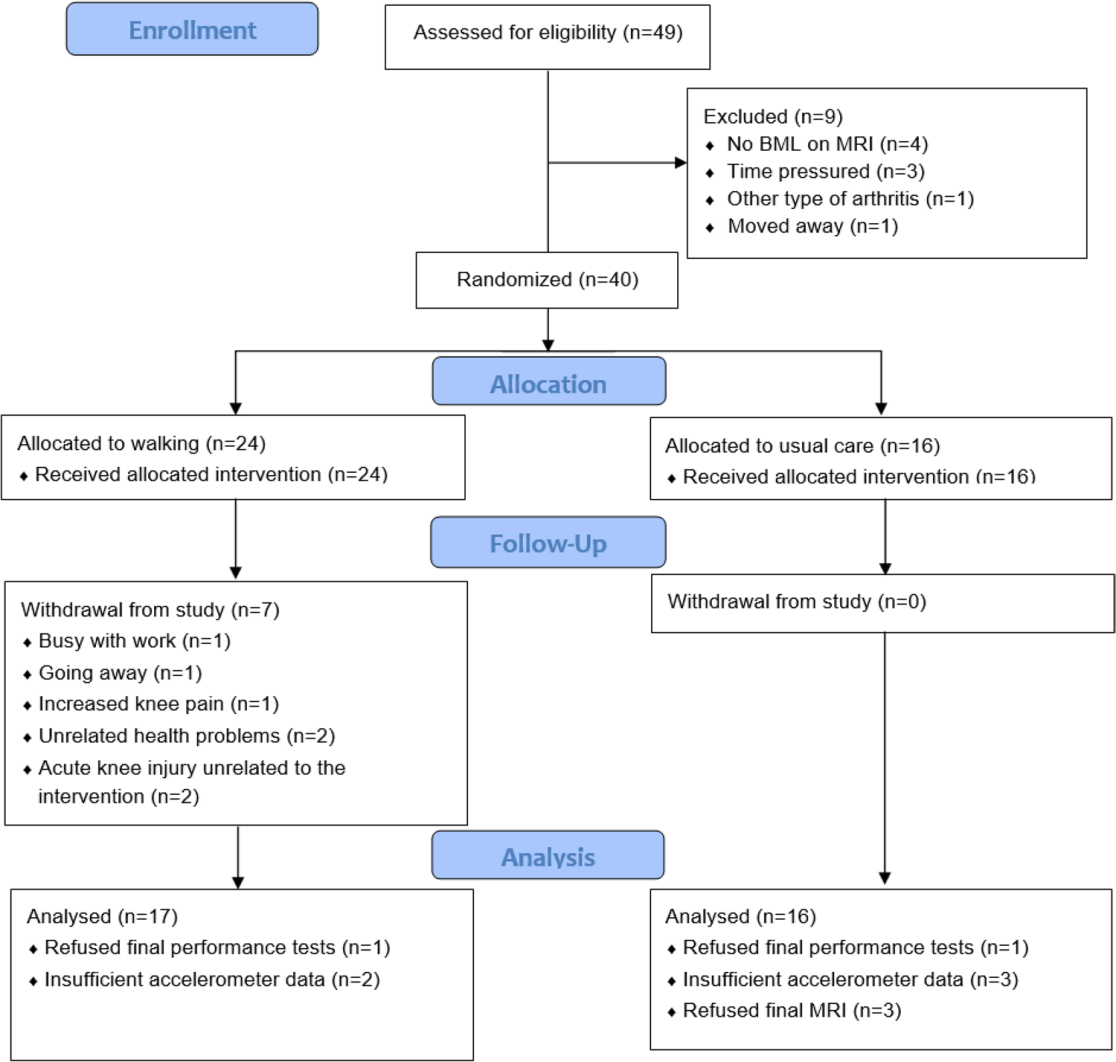
Flow diagram of participant recruitment and completion

Over 9 months, 49 potential participants were screened for eligibility of whom 40 (81.6%) were enrolled. They had a mean age of 66 years [SD 1.36], mean BMI of 32.9 kg/m^2^ (5.3), and 60% were female. Twenty-four participants were randomized to walking and 16 to the usual care group (Fig. 1). The characteristics of participants by group allocation are presented in Table 1.

**Table 1:** Baseline characteristics of participants

Baseline characteristics	*N*	Walking	*N*	Usual care	*p* value
Age, years: mean (SD)	24	65.1 (8.9)	16	67.4 (8.3)	0.416
Women: *n* (%)	24	15 (62.5)	16	9 (56.3)	0.693
BMI, kg/m^2^: mean (SD)	24	33.6 (4.9)	16	31.8 (5.8)	0.302
Waist/hip ratio: mean (SD)	24	0.9 (0.1)	16	0.9 (0.1)	0.812
Symptoms^a^					
VAS pain (0–100 mm): mean (SD)	24	55.3 (17.3)	16	52.2 (13.0)	0.812
WOMAC pain (0–500 mm): mean (SD)	24	250.0 (78.7)	16	242.3 (56.4)	0.735
WOMAC function (0–1700 mm): mean (SD)	24	915.8 (232.9)	16	893.4 (185.9)	0.894
WOMAC stiffness (0–200 mm): mean (SD)	24	107.4 (35.5)	16	113.4 (27.3)	0.571
Performance and strength					
30-s chair stand test^b^: score mean (SD)	24	11.0 (1.9)	16	11.3 (2.9)	0.785
40-m fast-paced walk test (m/s)^c^: mean (SD)	24	29.7 (4.7)	16	31.1 (6.4)	0.431
6-min walk test (m)^d^: score mean (SD)	24	478.5 (91.7)	16	472.1 (112.7)	0.844
Stair climb test (s)^e^: score mean (SD)	24	20.2 (9.1)	16	20.5 (10.7)	0.923
Timed up and go (s)^e^: score mean (SD)	24	9.5 (1.5)	16	9.8 (1.9)	0.604
Leg strength (kg)^f^: mean (SD)	24	71.0 (8.8)	16	60.2 (9.5)	0.422
Questionnaires					
PHQ-9 score (0–27)^g^: mean (SD)	24	4.4 (4.5)	16	4.8 (4.8)	0.801
EQ-5D-5L index value (0–1)^h^: mean (SD)	24	0.7 (0.0)	16	0.7 (0.0)	0.119
AQol-8D utility score (0–1)^i^: mean (SD)	24	0.8 (0.1)	16	0.8 (0.1)	0.185
Medication usage					
Paracetamol usage: *n* (%)	24	8 (33.3)	16	5 (31.3)	0.890
Paracetamol dose: mg: mean (SD)	8	1039.0 (330.0)	5	1065 (546.2)	0.939
COX-2 inhibitor usage: *n* (%)	24	0 (0)	16	2 (12.5)	0.076
NSAIDS usage: *n* (%)	24	9 (37.5)	16	6 (37.5)	0.999
Accelerometer measures^j^					
Sedentary minutes per week: mean (SD)	23	3830.6 (851.6)	16	3835.6 (508.5)	0.977
Light PA minutes per week: mean (SD)	23	1876.1 (485.0)	16	2022.4 (481.9)	0.283
MVPA minutes per week: mean (SD)	23	123.1 (97.9)	16	106.3 (103.8)	0.704
MRI measurements					
Total BML size (mm^2^): mean (SD)	23	357.0 (367.8)	16	391.0 (428.3)	0.789
Total effusion-synovitis size (ml): mean (SD)	24	47.5 (35.3)	16	45.2 (18.1)	0.808
Cartilage defect prevalence^k^: *n* (%)	24	22 (91.7)	16	15 (95.8)	0.870
Meniscal extrusion prevalence^l^: *n* (%)	24	15 (62.5)	16	9 (56.0)	
Radiographic measurements					
Alignment^m^: *n* (%)	23		16		
Normal alignment		3 (13.0)		1 (6.3)	0.519
Genu varus		16 (70.0)		14 (87.5)	0.136
Genu valgus		4 (17.4)		1 (6.3)	0.329
Osteophytes (0–12): mean (SD)^o^	23	2.4 (3.3)	16	1.7 (1.6)	0.410
Radiographic medial JSN^o^: *n* (%)	23		16		
Grade 0		8 (34.8)		3 (18.8)	0.274
Grade 1		4 (17.4)		7 (43.8)	0.073
Grade 2		5 (21.7)		2 (12.5)	0.460
Grade 3		6 (26.1)		4 (25)	0.939
Radiographic lateral JSN^o^: *n* (%)	23		16		
Grade 0		16 (60.6)		12 (75)	0.711
Grade 1		2 (8.7)		1 (6.3)	0.778
Grade 2		2 (8.7)		2 (12.5)	0.700
Grade 3		3 (13)		1 (6.3)	0.492

Statistics are presented as mean and standard deviation (SD) for continuous variables or *n* (proportion) for categorical variables. Abbreviations: *BMI*, body mass index; *COX*, cyclooxygenase; *NSAID*, non-steroidal anti-inflammatory drugs. ^a^Higher score in visual analogue scale (VAS) pain and WOMAC, Western Ontario and McMaster Universities Arthritis Index, indicate a more severe symptom. ^b^Number of stances made within 30 s. ^c^Walking speed in m/s. ^d^Meters walked within six minutes. ^e^Time in seconds. ^f^Force in KG. ^g^Patient health questionnaire (PHQ-9), a depression module in which a lower score is better. ^h^EURoQol-5 dimensions-5 levels (EQ5D-5L); index values were calculated using the UK data set, and a score closer to 1 is better. ^i^Assessment of quality of life (AQoL), utility scores were obtained by AQoL utility formulae, and a score closer to 1 is better. ^j^PA accelerometer, change in physical activity (PA) objectively measured by accelerometer including minutes in moderate to vigorous physical activity (MVPA). ^k^Defined as a cartilage defect present of grade 2 or greater. ^l^Defined as meniscal extrusions present of grade 1 or greater. ^m^Anatomic knee alignment based upon methods of Moreland et al.^28^. ^o^Mean cumulative grade of osteophytes and grade of joint space narrowing (JSN) determined utilizing the OARSI atlas^31^, a lower grade is better

The target sample size of 48 participants could not be reached due to budgetary constraints, and the process of simple randomization resulted in a difference in the number of participants randomized to each group. During the study, some changes to the protocol were required. Following the prolonged absence of a participant, availability was added as an exclusion criterion, excluding those who were unable to attend two class times per week or those who had planned absences (e.g., trips away) of > 2 weeks during the 24-week timeframe. Furthermore, at the walking sessions, participants would walk at different pasts making a group size of 10 challenging to supervise. Therefore, trainers requested to reduce group sizes to 6–8. Over the 24-week study period (Table 2), adherence was 70.0% to the total program of 72 scheduled walk sessions, 60.8% to the 48 scheduled group sessions, and 88.4% to the 24 unsupervised sessions. The total program adherence to 36 sessions over 12 weeks was better during weeks 0–12 than weeks 12–24 (85.1% for the first 12 weeks and 54.9% for the second 12 weeks). Meanwhile, a similar trend was observed in the attendance of weekly sessions by retained participants (90.0% for the first 12 weeks and 73.3% for the second 12 weeks). The retention rate at week 24 was 70.8% in the walking group and 100% in the usual care group.

**Table 2:** Adherence measures over the first 12 weeks (0–12) and the second 12 weeks (12–24) for those participants in the walking group (*n* = 24)

	Weeks 0–12		Weeks 12–24		Weeks 0–24	
	Mean (SD)	Adherence rate, %	Mean (SD)	Adherence rate, %	Mean (SD)	Adherence Rate, %
Over 12 and 24 weeks						
Total number of walks completed	30.6 (9.6)	85.1 (30.6/36)	19.8 (14.9)	54.9 (19.8/36)	50.4 (23.1)	70.0 (50.4/72)
Number of group walks completed	18.2 (6.9)	75.8 (18.2/24)	11.0 (9.3)	45.8 (11.0/24)	29.2 (14.9)	60.8 (29.2/48)
Number of home walks completed	12.4 (3.7)	103.3 (12.4/12)	8.8 (7.6)	73.3 (8.8/12)	21.2(10.2)	88.4 (21.2/24)
Per week						
Total number of walks per week	2.7 (0.6)	90.0 (2.7/3)	2.2 (0.9)	73.3 (2.2/3)	2.6 (1.1)	86.6 (2.6/3)
Number of group walks per week	1.6 (0.5)	80.0 (1.6/2)	1.3 (0.6)	65.0 (1.3/2)	1.5 (0.9)	75.0 (1.5/2)
Number of home walks per week	1.1 (0.3)	110.0 (1.1/1)	1.1 (0.5)	110.0 (1.1/1)	1.1 (0.8)	110 (1.1/1)

Safety was analyzed using adverse event rates which are shown in Table 3. At least 1 adverse event was reported by 12 (50%) participants in the walking group and 6 (38%) in the usual care group. Within the walking group, most adverse events were musculoskeletal (11 vs 6), while in the usual care group, the majority were non-musculoskeletal (4 vs 2). Adverse events that were deemed related to the walking intervention (*n* = 6) were classified as mild and included foot pain (*n* = 2), chest tightness (*n* = 1), dizziness (*n* = 1), shortness of breath (*n* = 1), and one fall incident (*n* = 1). Three serious adverse events occurred that were deemed unrelated to the study (walking group: pneumonia (*n* = 1); complications due to anemia (*n* = 1); usual care group: angioplasty (*n* = 1)). During screening, an error occurred while interpreting the accelerometer data, and despite the initial criteria to exclude individuals who met physical activity guidelines, 5 (31.3%) participants in the usual care group and 7 (29.2%) in the walking group who met these guidelines had not been excluded.

**Table 3:** Reported adverse events during the study

	No. (% of participants)		
	Walking (*N* = 24)	Usual care (*N* = 16)	*p* value
Adverse events			
Total number of adverse events	26	8	
At least one experienced	12 (50)	6 (38)	0.436
Any serious	2 (8)	1 (6)	0.806
Treatment related	6 (25)		0.030
Musculoskeletal	11	2	
Increased knee pain	3 (13)	1(6)	0.519
Foot pain	2 (8)		0.236
Upper body pain	3 (13)		0.141
Plantar fasciitis	1 (4)		0.408
Calf strain		1 (6)	0.215
Acute knee injury, unrelated	2 (8)		0.236
Non-musculoskeletal	6	4	
Knee VTE		1 (6)	0.215
Angioplasty		1 (6)	0.215
PMR		1 (6)	0.215
Anemia	1 (4)		0.408
Flu		1 (6)	0.215
Pneumonia	1 (4)		0.408
Bronchi	1 (4)		0.408
Chest infection	3 (13)		0.141

Abbreviations: *PMR*, polymyalgia rheumatica; *TKR*, total knee replacement; *VTE*, venous thromboembolism

### Exploratory results

**Fig. 2:**
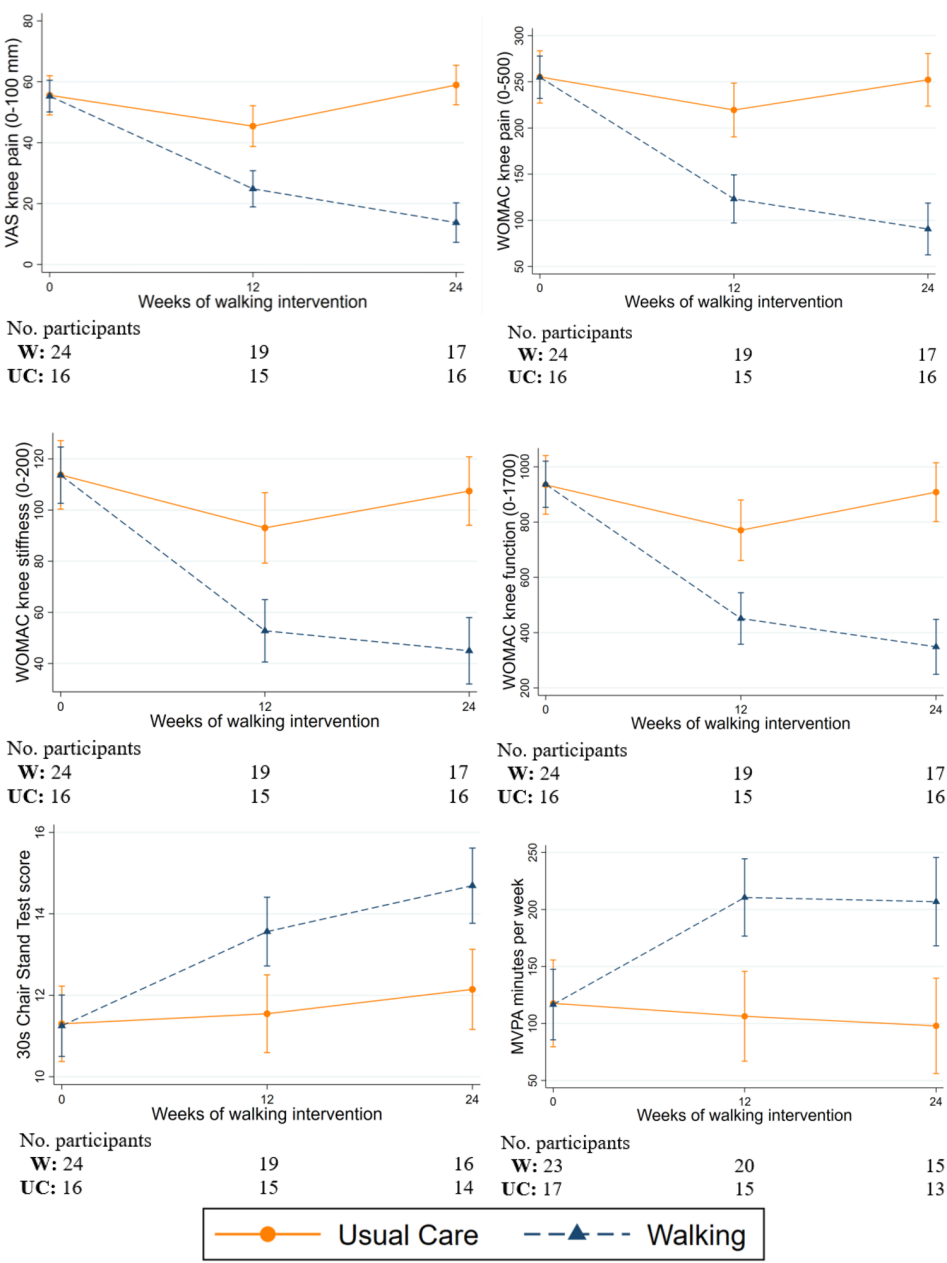
**VAS**, WOMAC subscales, 30-s chair stand test, and changes in time spent in MVPA values in the walking and usual care group during 24 weeks of intervention (mean with 95% confidence interval). The data are estimates from linear mixed-effects models, adjusted for age, sex, and corresponding baseline values. Abbreviations: MVPA, moderate to vigorous physical activity; VAS, visual analogue scale; WOMAC, Western Ontario and McMaster University Index; W, walking; UC, usual care

Exploratory results are presented in Table 4 and Fig. 2. Over 24 weeks, participants in the walking group had improved VAS knee pain, while those in the usual care group did not (change of − 38.7 mm [95% CI − 47.1 to − 30.3] and 4.3 mm [− 4.9 to 13.4], respectively). The walking group experienced greater mean improvements in WOMAC pain, function and stiffness, each OARSI performance measure, leg strength, and weekly time spent in MVPA and were 4 times more likely to meet the OMERACT-OARSI responder criteria compared to the usual care group. There are very small changes in MRI-based structural measures over 24 weeks in each group, with the magnitude and direction of effects shown in Table 4.

**Table 4:** Change in secondary outcomes over 24-week follow-up between the walking and usual care group

	Walking			Usual care			Between group difference	
	Baseline mean (SE)	24-weeks mean (SE)	Change mean (95% CI)	Baseline mean (SE)	24-week mean (SE)	Change mean (95% CI)	Change, mean (95% CI)	*p* value
Symptoms^a^								
VAS knee pain (0–100 mm)	55.3 (3.5)	16.6 (4.1)	− 38.7 (− 47.1 to − 30.3)	52.2 (4.3)	56.4 (4.3)	4.3 (− 4.9 to 13.4)	− 44.9 (− 56.6 to − 33.3)	< 0.0001
WOMAC pain (0–500 mm)	250.0 (15.6)	93.8 (17.8)	− 156.3 (− 191.6 to − 120.9)	242.3 (19.1)	244.3 (19.1)	2.0 (− 36.1 to 40.1)	− 161.3 (− 209.5 to − 113.0)	< 0.0001
WOMAC stiffness (0–200 mm)	107.4 (7.3)	43.6 (8.3)	− 63.8 (− 80.1 to − 47.5)	113.4 (9.0)	107.1 (9.0)	-6.3 (− 23.8 to 11.2)	− 62.4 (− 84.1 to − 40.6)	< 0.0001
WOMAC function (0–1700 mm)	915.8 (51.3)	350.1 (58.9)	− 565.6 (− 685.3 to − 446.0)	917.8 (64.3)	894.8 (62.8)	− 23.0 (− 154.8 to 108.9)	− 561.9 (− 729.5 to − 394.2)	< 0.0001
Performance and strength								
30-s chair stand test ^b^	11.0 (0.5)	14.7 (0.6)	3.6 (2.5 to 4.8)	11.3 (0.7)	12.1 (0.7)	0.8 (-0.4 to 2.1)	2.6 (1.0 to 4.2)	0.002
6-min walk test, (m)^c^	478.5 (19.8)	551.5 (20.9)	73.0 (47.0 to 99.0)	472.1 (24.3)	500.2 (24.7)	28.1 (-0.7 to 57.0)	48.0 (12.0 to 83.9)	0.009
40-m fast-paced walk test, (m/s)^d^	1.4 (0.0)	1.6 (0.1)	0.2 (0.2 to 0.3)	1.3 (0.1)	1.5 (0.1)	0.1 (0.1 to 0.2)	0.09 (− 0.01 to 0.18)	0.033
Stair climb test score, (s)^e^	20.2 (1.5)	14.4 (1.7)	− 5.8 (− 8.6 to − 3.1)	20.5 (1.9)	18.4 (1.9)	− 2.6 (− 5.6 to 0.4)	− 3.7 (− 6.0 to − 1.3)	0.002
Timed up and go, (s)^e^	9.5 (0.3)	8.6 (0.3)	− 0.9 (− 1.4 to − 0.4)	9.8 (0.4)	9.3 (0.4)	− 0.5 (− 1.0 to 0.3)	− 0.60 (− 1.23 to 0.04)	0.066
Leg strength, (kg)^f^	71.0 (7.7)	86.1 (8.2)	15.2 (3.8 to 26.5)	60.2 (9.4)	71.9 (9.7)	11.7 (− 1.3 to 24.7)	8.4 (− 6.5 to 23.2)	0.268
Questionnaires								
PHQ-9 (0–27)^g^	4.4 (0.8)	3.1 (0.9)	− 1.2 (− 2.7 to 0.2)	4.8 (1.0)	4.9 (1.0)	0.1 (− 1.5 to 1.7)	− 1.7 (− 3.6 to 0.1)	0.071
AQoL utility score (0–1)^h^	0.81 (0.02)	0.86 (0.02)	0.05 (0.02 to 0.07)	0.78 (0.03)	0.78 (0.03)	− 0.001 (− 0.03 to 0.03)	0.06 (0.02 to 0.10)	0.002
EQ5D-5L, index value (0–1)^i^	0.73 (0.02)	0.74 (0.02)	0.02 (− 0.03 to 0.06)	0.69 (0.03)	0.70 (0.03)	0.01 (− 0.04 to 0.06)	0.02(− 0.05 to 0.09)	0.520
PA accelerometer^j^								
Sedentary minutes per week	3836.4 (152.2)	3880.4 (177.2)	44.1 (− 229.5 to 367.6)	3841.9 (186.4)	3896.8 (198.6)	− 54.5 (− 302.8 to 411.9)	165.8 (− 273.0 to 604.6)	0.459
Light PA minutes per week	1854.8 (96.2)	1840.7 (107.3)	− 14.1 (− 180 to 152.2)	2027.9 (117.8)	2155.0 (123.2)	127.0 (− 55.8 to 309.9)	− 44.4 (− 309.8 to 221.1)	0.743
MVPA minutes per week	119.6 (24.8)	209.0 (28.2)	89.5 (42.0 to 137.0)	107.1 (30.4)	90.2 (32.0)	-16.9 (-69.3 to 35.4)	109.9 (42.6 to 177.3)	0.001
Structure changes								
BMLs, mm^2^	357.0 (76.0)	320.6 (77.8)	− 36.5 (− 100.2 to 27.3)	391.7 (91.1)	356.6 (92.5)	− 35.1 (− 108.0 to 37.9)	− 1.1 (− 72.4 to 74.5)	0.977
Effusion-synovitis, ml	47.5 (6.0)	47.6 (6.0)	0.1 (− 5.8 to 6.0)	45.2 (70.5)	48.4 (72.0)	3.3 (− 3.5 to 10.1)	− 2.4 (− 9.8 to 4.9)	0.515
Cartilage defects^k^			Proportion, *n* (%)			Proportion, *n* (%)	Risk ratio (95% CI)	
Increase			6 (35.3%)			6 (46.2%)	0.8 (0.3 to 1.8)	0.546
Stable			9 (52.9%)			7 (53.8%)	1.0 (0.5 to 1.9)	0.961
Decrease			2 (11.8%)			0 (0.0%)	-	
Meniscal extrusions^l^								
Increase			4 (23.5%)			1 (7.7%)	3.1 (0.4 to 24.2)	0.290
Stable			13 (76.5%)			11 (84.6%)	1.0 (0.7 to 1.5)	0.977
Decrease			0 (0.0%)			1 (7.7%)	-	
OMERACT-OARSI responders^m^			17 (100%)			4 (25%)	4.0 (1.7 to 9.3)	0.001

Abbreviations: *CI*, confidence interval; *VAS*, visual analogue scale; *WOMAC*, Western Ontario and McMaster University Index; *OARSI-OMERACT*, Osteoarthritis Research Society International-Outcome Measures in Rheumatology Clinical trials; *AQoL*, assessment of quality of life; *MVPA*, moderate to vigorous physical activity; *BML*, bone marrow lesion. ^a^Higher score in VAS pain or WOMAC outcomes indicates a more severe symptom. ^b^Number of stances made in 30 s. ^c^Meters walked within 6 min. ^d^Walking speed in meter per second. ^e^Time in seconds. ^f^Force in kilogram. ^g^PHQ-9 is a depression module, and lower score is better. ^h^AQoL utility scores were obtained by AQoL utility formulae using four of the five dimensions of the assessment of quality of life questionnaire. ^*i*^*EQ5D-5L*, EURoQol-5 dimensions-5 levels; index values were calculated using the UK data set. ^j^PA accelerometer, physical activity objectively measured by accelerometer. ^k^Defined as a cartilage defect present of grade 2 or greater. ^l^Defined as meniscal extrusions present of grade 1 or greater. ^m^OMERACT- OARSI responders (0 = non-responder, 1 = responder). For continuous variables, baseline, 24-week and change values were derived from unadjusted mixed-effects models. The between group differences were derived from mixed effects models adjusted for age, sex, and corresponding baseline values. For the analyses of categorical variables log binomial regression was used

## Discussion

This pilot demonstrates feasibility and lays the groundwork for a full-scale RCT that investigates the joint structural implications of a walking exercise program for KOA, though the recruitment approach could be improved. A subsequent full-scale RCT has the potential to build evidence about the clinical benefit of exercise therapy and help to debunk common misconceptions that exercise causes joint harm [7].

The study demonstrates feasibility in terms of randomization, safety, retention, and adherence and presents improvements to the study design. Supervised outdoor walking allowed for individualized support and encouragement for participants, attributing to high patient satisfaction and symptomatic improvements. Group sizes were reduced to provide better individualized support and to ensure protocol adherence and safety. In addition, adding planned absences as an exclusion criterion helped to promote adherence to the program.

Overall adherence to the walking program was 70.0%, which is higher than most comparable exercise studies [8]. The total program adherence was better during the first 12 weeks (85.1%) compared to the second 12 weeks (54.9%), and increased adherence promotion strategies during the second half of the study could further increase the quality of a more definitive study. In participants who remained in the study for the full 24 weeks, attendance remained high for the whole study duration (90.0% for the first 12 weeks and 73.0% for the second 12 weeks). The overall retention rate within the walking group (70.8%) was equal to [34, 35] or lower than [9] comparable studies. Dominant reasons for exclusion and withdrawal included being busy with work and going away. Prioritizing the screening of participants on time availability and making verbal agreements to complete follow-up measurements at the least could potentially benefit enrollment, adherence, and retention. This study did not investigate whether adherence and retention were related to the amount of benefit participants were experiencing and this is an area of future research which could potentially be better understood using a qualitative component. The intervention was considered safe. Six adverse events were deemed possibly related to the intervention and these were mild in nature and could be managed by the supervising physiotherapist and/or exercise physiologist.

Recruitment was slower than anticipated, and the recruitment target could not be reached due to budgetary constraints. This could have been due to our institute recruiting for two KOA exercise trials simultaneously, and this will be considered when planning the implementation of a future large-scale trial. Recruitment in future studies could potentially benefit from an enhanced recruitment strategy, such as snowball sampling, engaging primary care practitioners, promotion through local organizations (e.g., Arthritis & Osteoporosis Tasmania), and by including more walk locations or using a multi-center design. The use of simple randomization resulted in an imbalance between the number of participants in each group, which could be prevented in a future trial by using block randomization [36].

This pilot study investigated the direction and magnitude of changes in several exploratory outcomes. As this was a pilot study, no a priori power estimations were performed. Walking improved knee pain, function, and stiffness over 24 weeks, which supports the findings of previous RCTs [37]. The improvement in VAS pain observed in the walking group (− 38.7; (95% CI − 47.1 to − 30.3) is clinically important [38]. There was also a mean clinically important improvement seen in WOMAC function (− 565.6 mm (95% CI − 685.3 to − 446.0)) [38], and all walking group participants met the OMERACT-OARSI responder criteria [22]. Some factors could have contributed to the relatively large improvements in symptoms. One is that all participants who completed the walking intervention reported to be extremely satisfied. Patient satisfaction is a contextual effect rather than a direct treatment effect, which can be responsible for 5% (− 10 to 33%) of the measured effect [39]. In addition, symptoms were self-reported by participants, who were not blinded, and this could contribute to an overestimation of benefits. Self-reported quality of life improved more in the walking group as well, and the average improvement in the walking group approached clinical importance for the AQoL scale (the minimum important difference in AQoL scores for the Australian population is 0.06 [40]).

Physical performance and activity were assessor-blinded measures conducted to objectively examine changes in the ability to perform daily activities. Both the walking and control group showed improvements in OARSI performance measures over 24 weeks, which may have been due to a test learning effect [41]. However, improvements in the walking group were larger for every test. At baseline, most participants did not meet physical activity guidelines of 150 min spent in MVPA per week. During the intervention, most participants in the walking group met the MVPA guidelines. This is important, because adults above 60 years who are physically active have a reduced risk of cardiovascular and all-cause mortality, cancer, fractures, recurrent falls, functional limitations, cognitive diseases, and a better quality of life [42].

There were very small changes in MRI-based structural measures over 24 weeks in each group. Apart from a slight difference in meniscal extrusion score increases between the groups, there was no indication of detrimental effects related to the walking intervention. Meniscal extrusions increased by 23.5% in the walking group and 7.7% in the control group. This finding was based on a small number of participants (*n* = 5) and needs to be verified in a larger study.

This study has several strengths. First, its design as a pilot RCT enabled a thorough investigation of the study design to improve the quality for a subsequent larger RCT. Second, the intervention was intensively supervised ensuring standardization of exercise and reducing self-report bias of exercise duration and frequency [43]. Third, the strategies to enhance participant satisfaction were a strength as this has shown to benefit retention and adherence [44]. Limitations of the study included a screening error meaning that twelve participants were included who met MVPA guidelines at baseline. Our original intention was to enroll KOA patients who were not very active, and this error could have diluted the benefit of the program or may propose an additional challenge to recruitment in a subsequent RCT. In addition, the different withdrawal rate between groups could be a source of potential attrition bias [45].

## Conclusion

A full-scale RCT is considered feasible given acceptable adherence, retention, randomization, and safety though its quality can be enhanced using the findings of this pilot. The large improvements in symptoms in the walking group support the potential clinical usefulness of a subsequent trial. Small changes were observed in MRI knee joint structure, and the estimates can be used to inform a more definitive study.

## Supplementary Information

Below is the link to the electronic supplementary material.

Supplementary file1 (DOCX 2044 KB)

Supplementary file2 (DOCX 18 KB)

## Data Availability

The data generated from this study will not be deposited in a public repository due to privacy and consent restrictions. De-identified data can be made available from the corresponding author on reasonable request, subject to a data sharing agreement.
