# Supplementary material for: A pilot randomized controlled trial evaluating outdoor community walking for knee osteoarthritis: walk
Source: Clin Rheumatol. 2023 Jan 24;42(5):1409–21. doi: 10.1007/s10067-022-06477-5 (PMC10102100; doi:10.1007/s10067-022-06477-5)
Supplement: Supplementary file 1 — Supplementary file1 (DOCX 2044 KB) [file 10067_2022_6477_MOESM1_ESM.docx]

**Appendix A.**

**WALK - WARM UP**

**In preparation for walk:** Walk at a medium pace 3-5 mins, then move through some range of motion drills and stretches for major walking muscle groups.

| 1. **Trunk and upper limbs**   Relaxed arm swings around trunk with trunk rotations.  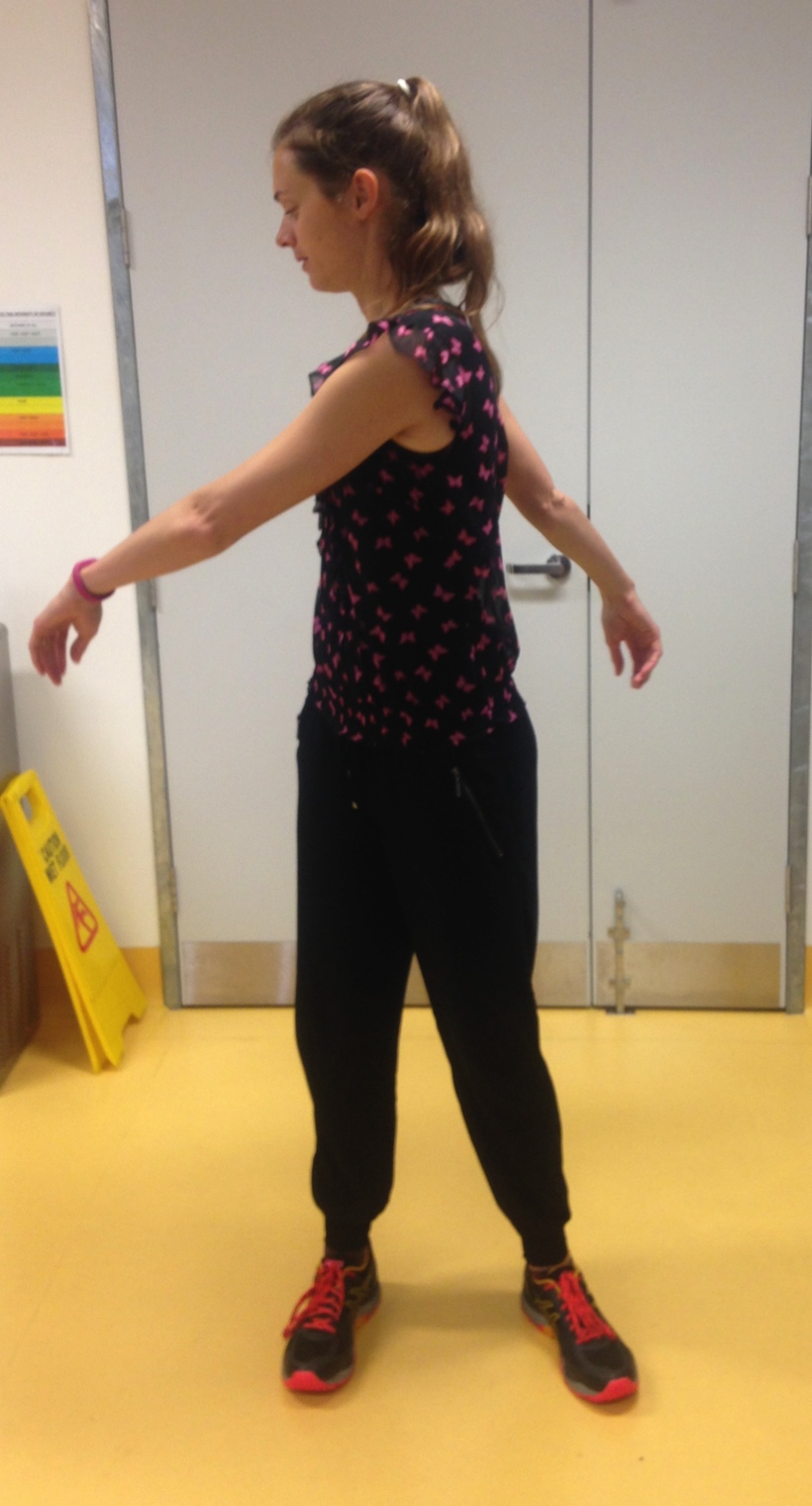 | 1. **Hip Adductors**   Feet wide, facing forward, moving between side lunge left and right. Finish with 1-2 short 5-10 sec holds on each side.  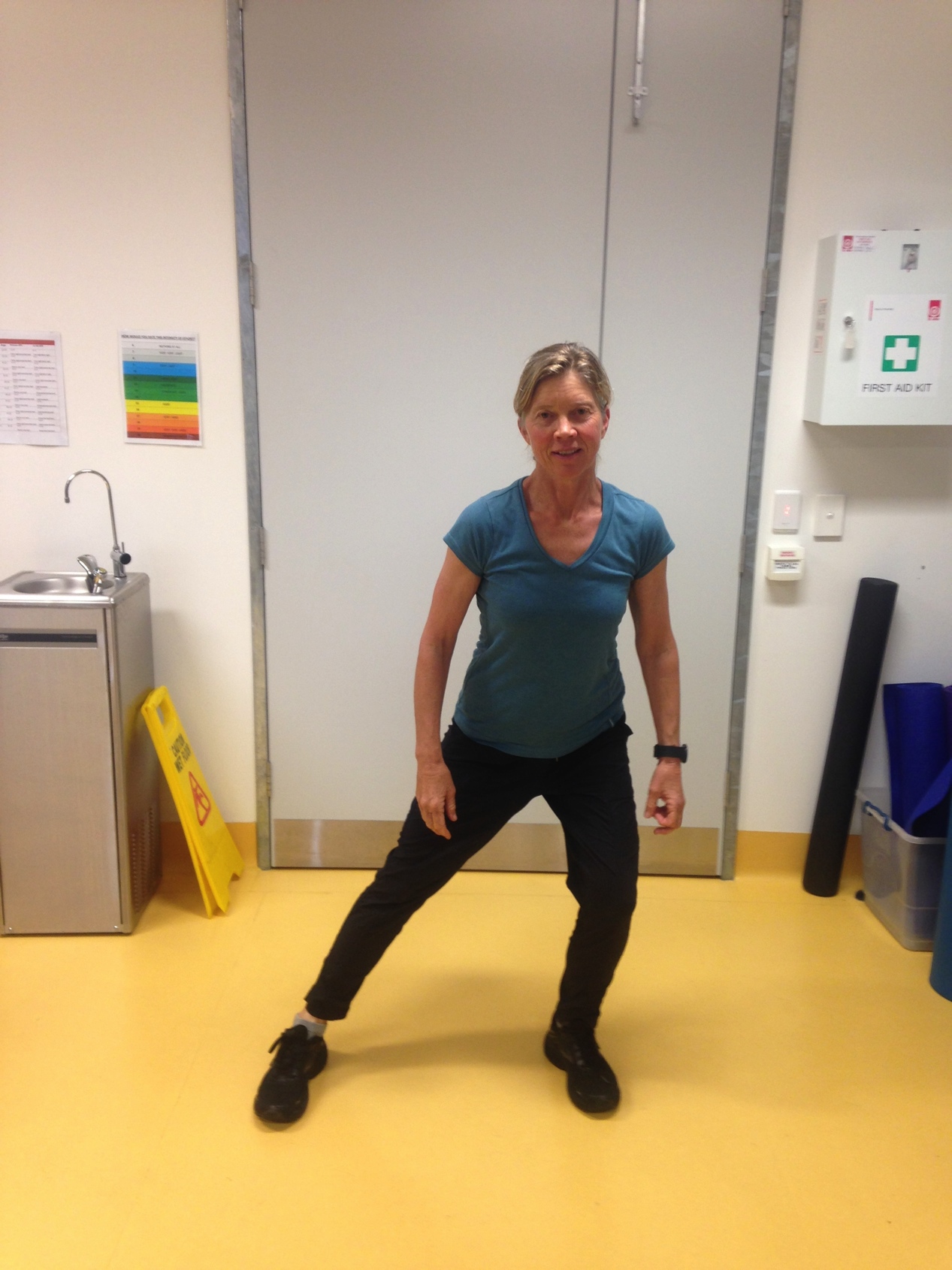 | 1. **Quadriceps/ Hamstring and Calf**   Long stride stance. Transfer weight from back leg to front leg. Finish with holds 5-10 sec, repeat other side.  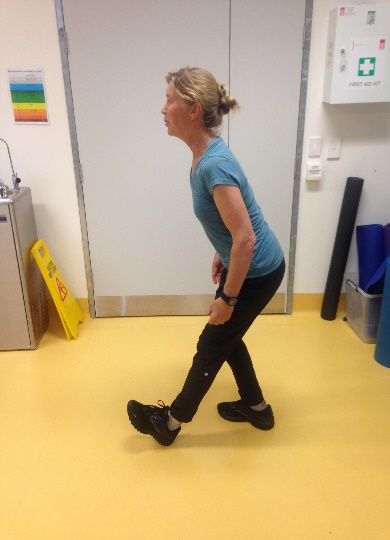 ↔ 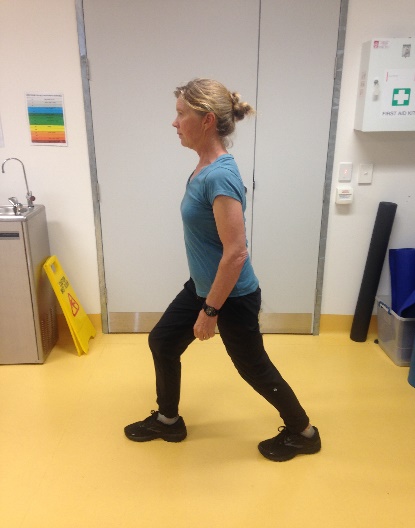 |
| --- | --- | --- |
| 1. **Hip flexor**   Upright stride stance with anterior pelvic tilt 5—10 sec hold.  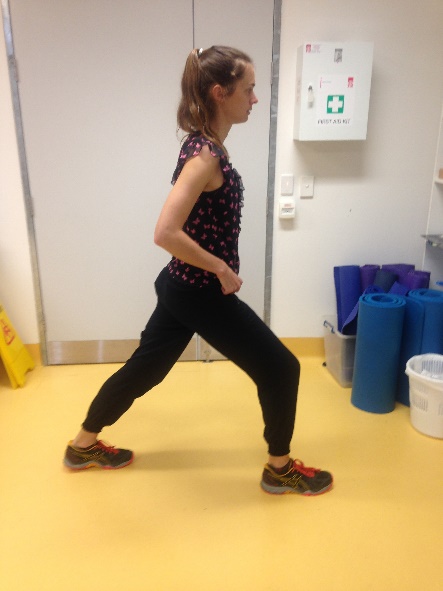 | 1. **Ankle pumps**    1. each leg- with support if needed   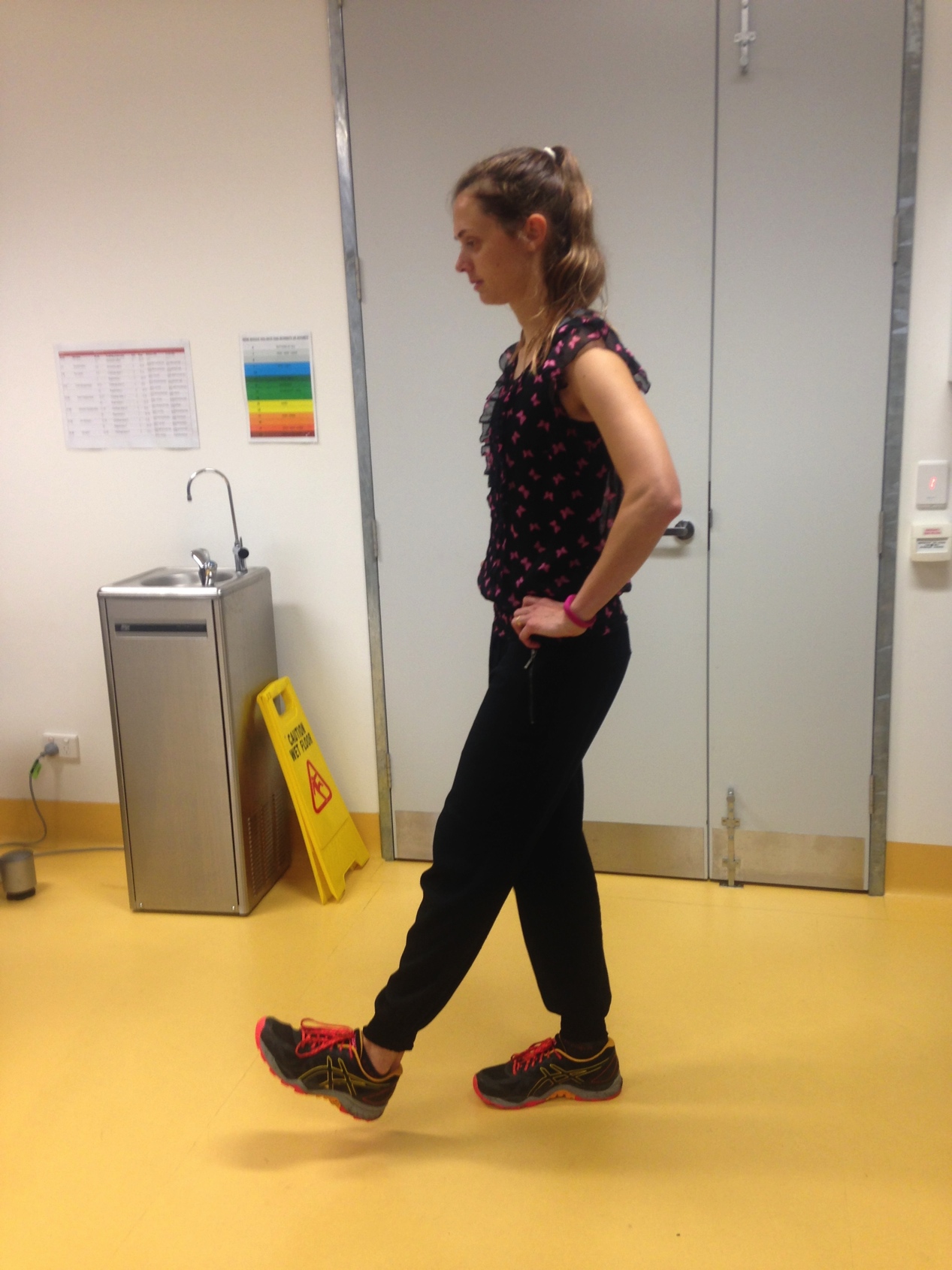 | 1. **Arm circles** |

**COOL DOWN / STRETCH**

Whole body cool down with sustained stretches – hold up to 30 seconds for each.

| 1. Diagonal whole body stretch – reaching above head   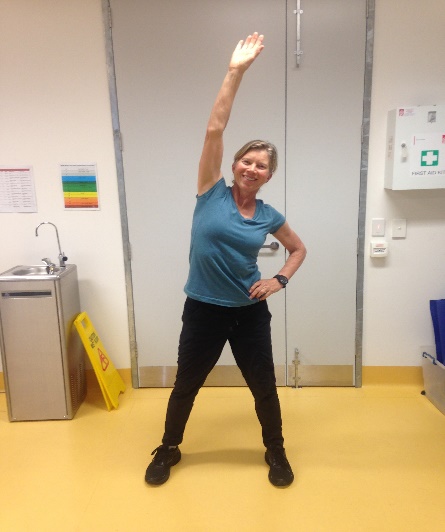 | 1. Stand tall/Pectorals and upper limb lengthening – hands behind back   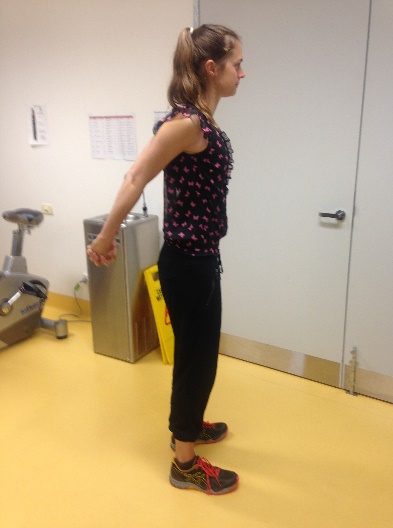 | 1. Upper trunk / upper limb   Rounding upper trunk and arms- ‘hug a tree’  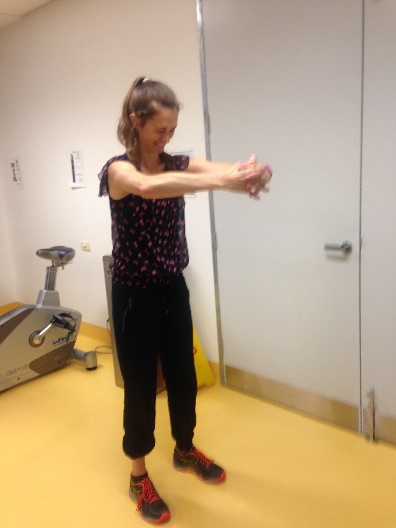 | 1. Adductor stretch in wide stance   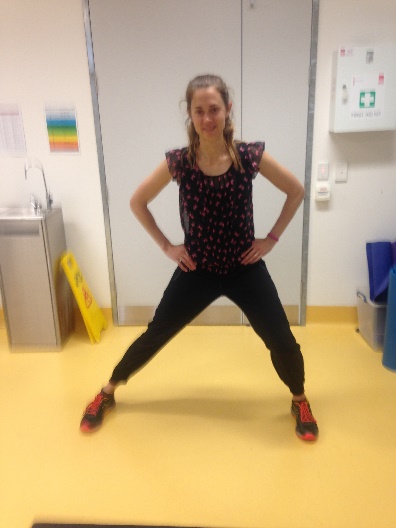 |
| --- | --- | --- | --- |
| 1. Supported stretch for Quadriceps   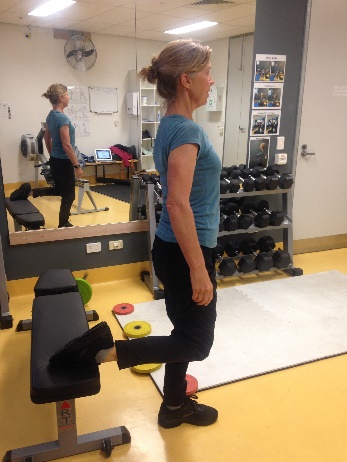 OR 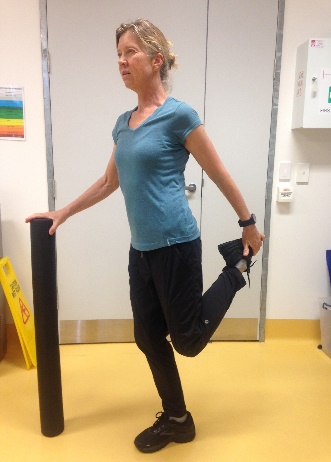 | 1. Supported stretch for Hamstrings   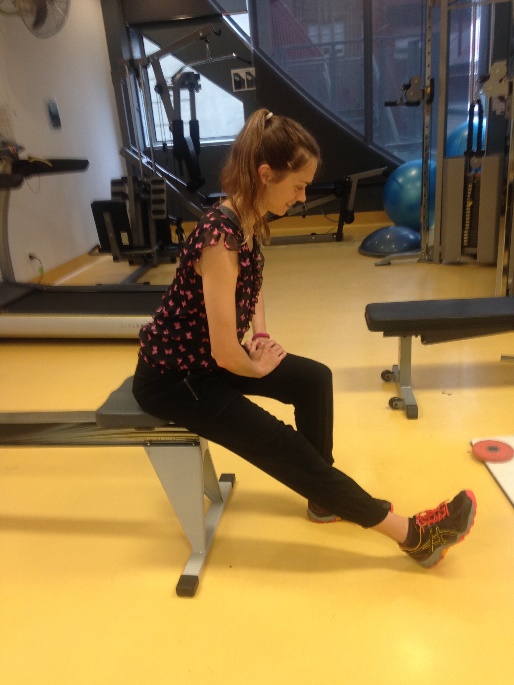 | 1. Calf stretch- Gastrocnemius   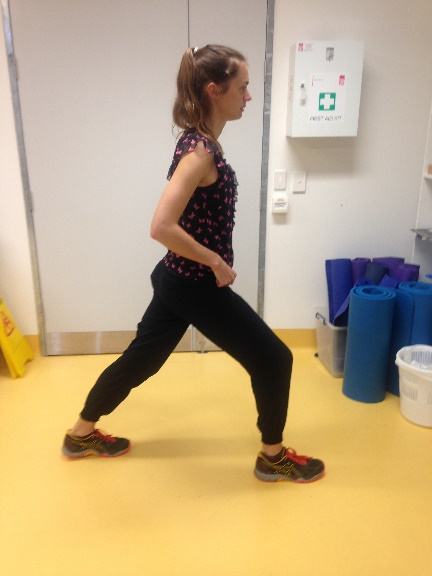 | 1. Calf stretch- Soleus   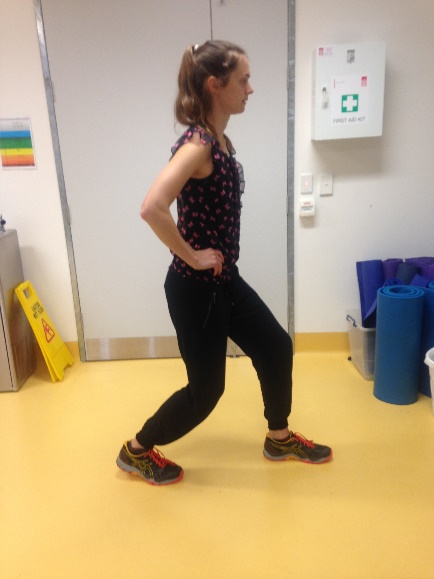 |
