# Supplementary material for: A pilot randomized controlled trial evaluating outdoor community walking for knee osteoarthritis: walk
Source: Clin Rheumatol. 2023 Jan 24;42(5):1409–21. doi: 10.1007/s10067-022-06477-5 (PMC10102100; doi:10.1007/s10067-022-06477-5)
Supplement: Supplementary file 2 — Supplementary file2 (DOCX 18 KB) [file 10067_2022_6477_MOESM2_ESM.docx]

**Appendix B**

| Supplementary Table 1. Magnetic imaging sequences and parameters | |
| --- | --- |
| **Sequence** | **Parameters** |
| T1-weighted fat-saturated 3D gradient-recalled acquisition | sagittal plane, flip angle 30 degrees; repetition time 38 msec; echo time 3 msec; field of view 16 cm; 512 × 512 matrix; 1 excitation; slice thickness 1.5 mm. |
| Proton density fat-saturated 2D fast spin echo sequence | sagittal plane flip angle 150 degrees; repetition time 3,800 msec; echo time 35 msec; field of view 16 cm; 512 × 512 matrix; 3 excitations; slice thickness 3 mm. |
